# Rice stripe virus coat protein induces the accumulation of jasmonic acid, activating plant defence against the virus while also attracting its vector to feed

**DOI:** 10.1111/mpp.12995

**Published:** 2020-09-24

**Authors:** Kelei Han, Haijian Huang, Hongying Zheng, Mengfei Ji, Quan Yuan, Weijun Cui, Hehong Zhang, Jiejun Peng, Yuwen Lu, Shaofei Rao, Guanwei Wu, Lin Lin, Xuemei Song, Zongtao Sun, Junmin Li, Chuanxi Zhang, Yonggen Lou, Jianping Chen, Fei Yan

**Affiliations:** ^1^ State Key Laboratory for Managing Biotic and Chemical Threats to the Quality and Safety of Agro‐products Institute of Plant Virology Ningbo University Ningbo China; ^2^ Key Laboratory of Biotechnology in Plant Protection of MOA and Zhejiang Province Zhejiang Academy of Agricultural Sciences Hangzhou China; ^3^ College of Plant Protection Nanjing Agricultural University Nanjing China; ^4^ School of Medicine Ningbo University Ningbo China; ^5^ State Key Laboratory of Rice Biology Institute of Insect Sciences Zhejiang University Hangzhou China

**Keywords:** coat protein, jasmonic acid, *Laodelphax striatellus*, rice stripe virus, small brown planthopper (SBPH)

## Abstract

The jasmonic acid (JA) pathway plays crucial roles in plant defence against pathogens and herbivores. Rice stripe virus (RSV) is the type member of the genus *Tenuivirus*. It is transmitted by the small brown planthopper (SBPH) and causes damaging epidemics in East Asia. The role(s) that JA may play in the tripartite interaction against RSV, its host, and vector are poorly understood. Here, we found that the JA pathway was induced by RSV infection and played a defence role against RSV. The coat protein (CP) was the major viral component responsible for inducing the JA pathway. Methyl jasmonate treatment attracted SBPHs to feed on rice plants while a JA‐deficient mutant was less attractive than wild‐type rice. SBPHs showed an obvious preference for feeding on transgenic rice lines expressing RSV CP. Our results demonstrate that CP is an inducer of the JA pathway that activates plant defence against RSV while also attracting SBPHs to feed and benefitting viral transmission. This is the first report of the function of JA in the tripartite interaction between RSV, its host, and its vector.

## Graphical abstract

Rice stripe virus coat protein is an inducer of the jasmonic acid pathway that activates plant defence against the virus while also attracting its vector to feed and benefitting viral transmission.

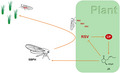

Jasmonic acid (JA) is an oxygenated fatty acid (oxylipin) synthesized from α‐linolenic acid. It is one of the crucial plant hormones, regulating a wide range of processes, including growth, photosynthesis, reproductive development, and plant responses to biotic and abiotic stresses. It has been well established that JA positively regulates plant defence against herbivores and necrotrophic pathogens (Zhang *et al*., 2017). Its function in regulating plant defence to viruses is not well known (Alazem and Lin, 2015). In compatible plant–virus interactions, JA seems to positively regulate plant defence. For example, the JA pathway was induced while the brassinosteroid (BR) pathway was suppressed in rice black‐streaked dwarf virus (RBSDV)‐infected plants (He *et al*., 2017). Further studies with *coi1‐13* and *Go* mutants indicated that JA‐mediated defence can suppress the BR‐mediated susceptibility to RBSDV infection (He *et al*., 2017). Additionally, JA treatment enhanced plant resistance to coinfection by potato virus X (PVX) and potato virus Y (PVY), and tomato spotted wilt virus (TSWV) at the early stage of infection, enhanced systemic resistance to tobacco mosaic virus (TMV), and disrupted geminivirus infection (Lozano‐Durán *et al*., 2011; Garcia‐Marcos *et al*., 2013; Zhu *et al*., 2014). These results support the view that JA positively regulates plant defence against viruses during compatible plant–virus interactions (Alazem and Lin, 2015). However, the role of JA in some incompatible plant–virus interactions may be negative. It has been shown that *N*‐mediated resistance to TMV was enhanced in the *NtCOI1*‐RNAi line or allene oxide synthase (*AOS*)‐silenced plants, and exogenous application of methyl jasmonate (MeJA) reduced local resistance to TMV, implying that JA negatively regulated *N*‐mediated resistance to TMV (Oka *et al*., 2013).

JA is generally considered to be one of the most important manipulators of plant defence against herbivores. Following insect attacks, plant pattern‐recognition receptors (PRRs) perceive herbivore‐associated molecular patterns (HAMPs) and damage‐associated molecular patterns (DAMPs), resulting in JA signalling‐dependent resistance (Dar *et al*., 2015). Caterpillars, thrips, aphids, leafhoppers, fungal gnats, and whiteflies all grow less well on plants treated with JA or its derivatives, indicating that JA plays critical roles in plant defence against a broad range of insects (Thaler *et al*., 2001; Boughton *et al*., 2006; Howe and Jander, 2008; Zhang *et al*., 2017). In contrast, *Nilaparvata lugens* show a preference for settling on JA‐treated plants (Lou *et al*., 2005). In addition, Zhou *et al*. (2009) found that silencing a critical JA biosynthesis gene (*OsHI‐LOX*) significantly enhanced rice resistance to *N. lugens*, indicating that JA‐related defence mechanisms are more complex than expected. Indeed, as an important defensive phytohormone, JA is the focus of competition in the coevolutionary arms race between plants and herbivores (Howe and Jander, 2008).

Rice stripe virus (RSV), the type member of the genus *Tenuivirus*, is transmitted by the small brown planthopper (SBPH), *Laodelphax striatellus*, and causes serious epidemics in East Asia (Wang *et al*., 2008). The role(s) played by JA in the tripartite interaction among RSV, its host, and its vector have not been studied. Here, we found that the JA pathway was induced by RSV infection and played a defence role against RSV. Interestingly, MeJA treatment attracted SBPHs to feed on rice plants. We conclude that CP is an inducer of the JA pathway that activates plant defence against RSV while also attracting SBPH to feed and benefitting viral transmission.

To determine if the JA signalling pathway was induced following RSV infection, quantitative reverse transcription PCR (RT‐qPCR) was used to detect the expression levels of JA synthesis genes, as described before (He *et al*., 2017). The expression levels of JA synthesis genes *Os13LOX*, *OsAOS*, *OsAOC*, and *OsOPR7* were determined in RSV‐infected *Oryza sativa* at 21 days postinfection (dpi) when stripe symptoms of RSV had become fully developed. In RSV‐infected plants the four genes were all up‐regulated compared with the mock‐inoculated control plants (Figure 1a). The expression of their homologs (*NbLOX*, *NbAOS*, *NbAOC*, and *NbOPR3*, respectively) were also up‐regulated in RSV‐infected *Nicotiana benthamiana* (an experimental host of RSV) at 14 dpi when symptoms of RSV had fully developed (Figure 1b). Consistent with the transcriptional levels of JA synthesis genes, the JA concentrations in RSV‐infected rice and *N. benthamiana* were significantly up‐regulated when compared with that of the controls (Figure 1c,d). All the results indicate that the JA pathway was induced by RSV infection in both rice and *N. benthamiana*.

**FIGURE 1:**
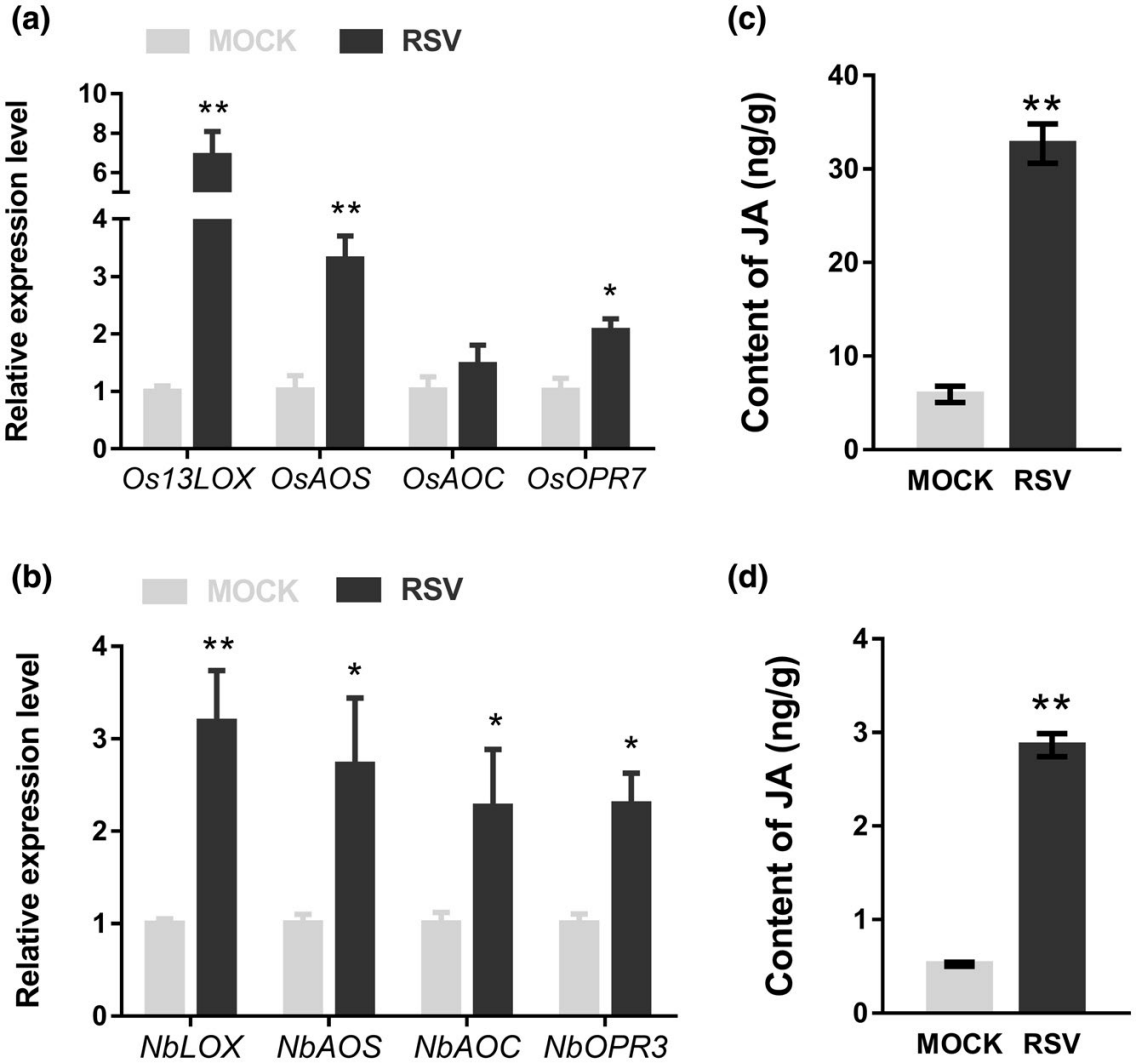
The jasmonic acid (JA) pathway is induced by rice stripe virus (RSV) infection. Relative expression levels of genes in the JA pathway in RSV‐infected rice (a) and *Nicotiana benthamiana* (b). JA content in RSV‐infected rice (c) and *N. benthamiana* (d). The *Actin* (*OsActin* or *NbActin*) gene was used as an internal control. Bars represent the standard errors of the means from three biological repeats. A two‐sample unequal variance directional *t* test was used to test the significance of the difference (*p < .05, **p < .01). ng/g is JA amount (ng) in leaves (fresh weight, g)

To investigate the potential roles of the JA pathway in RSV infection, we treated rice plants with MeJA (an integral component of the JA pathway) or salicylhydroxamic acid (SHAM, an inhibitor of the JA pathway), and monitored RSV symptom development and incidence on the treated plants. Plants were inoculated with RSV using SBPHs as described before (Tong *et al*., 2017). To avoid the effects of MeJA and SHAM on transmission of RSV by SBPHs, MeJA and SHAM were sprayed onto rice seedlings after the viruliferous SBPHs had fed on them. At 25 dpi, MeJA‐treated plants had less severe stripe symptoms, while SHAM‐treated plants had aggravated symptoms and an increased incidence of infection (Figure 2a,b). Northern and western blotting results showed that viral RNA and coat protein (CP) accumulated much less in MeJA‐treated plants but significantly more in SHAM‐treated plants (Figure 2c). In similar experiments on *N. benthamiana*, RSV symptoms were also less severe on MeJA‐treated plants (mild curling and mosaic on the upper leaves) compared to those on 0.1% ethanol‐treated control plants (Figure 2d). In contrast, more serious symptoms of RSV appeared on SHAM‐treated plants (Figure 2d). Northern and western blotting analyses showed that viral RNA and CP accumulated much less in either inoculated leaves or systemically infected leaves of MeJA‐treated plants, but significantly more in SHAM‐treated plants (Figure 2e). All the results indicate that JA plays a defence role against RSV in both rice and *N. benthamiana*.

**FIGURE 2:**
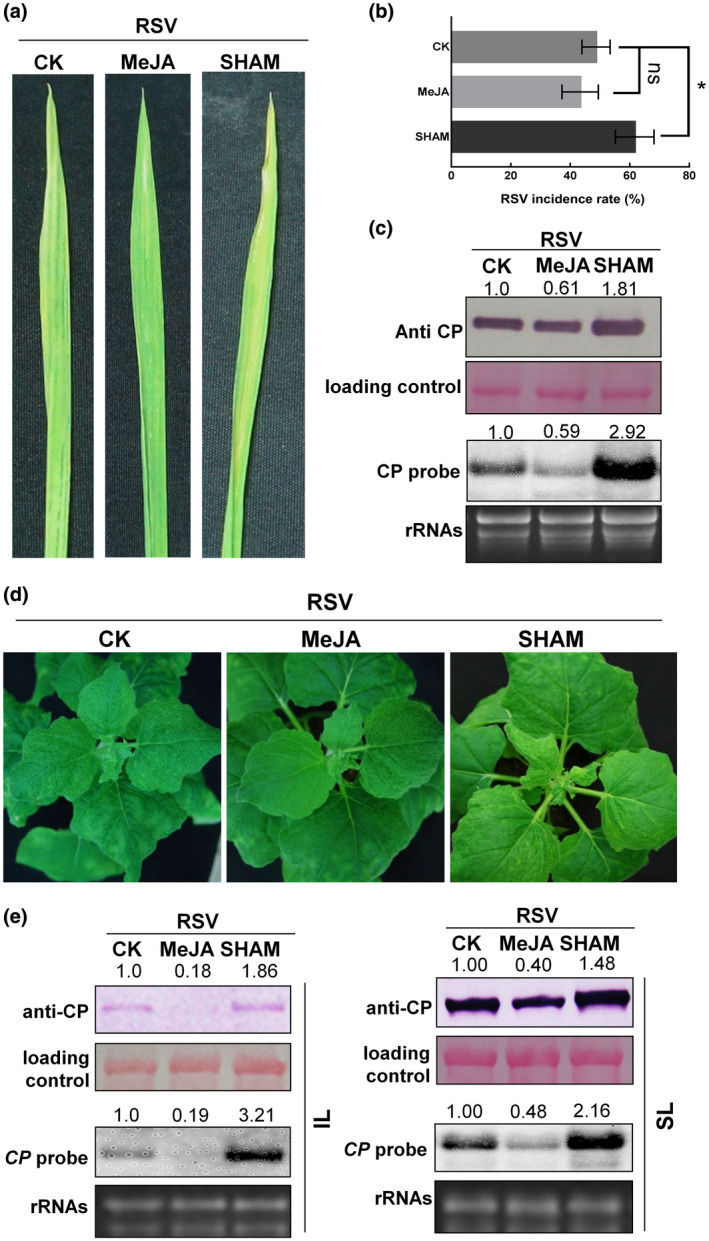
The jasmonic acid (JA) pathway plays a defence role against rice stripe virus (RSV). (a) RSV symptoms on rice plants treated with methyl jasmonate (MeJA) and salicylhydroxamic acid (SHAM) (an inhibitor of the JA pathway) at 25 days postinfection (dpi). 0.1% ethanol was used as the control (CK). (b) The incidence of RSV on MeJA‐, SHAM‐, and 0.1% ethanol (CK)‐treated rice plants at 25 dpi. The percentage of plants infected with RSV (incidence) was determined by reverse transcription‐PCR at 25 dpi. Error bars show the mean ± *SD* of three replicates (at least 30 plants per replicate). A two‐sample unequal variance directional Student's *t* test was used to test the significance of the differences (*p < .05; n.s., not significant). (c) Western and northern blotting results showing the accumulation levels of RSV coat protein (CP) and viral RNA, respectively, in rice plants treated with MeJA or SHAM. (d) RSV symptoms on *Nicotiana benthamiana* plants treated with MeJA or SHAM. (e) Western and northern blotting results showing the accumulation levels of RSV CP and viral RNA, respectively, in the inoculated leaves (IL) and systemically infected leaves (SL) of *N. benthamiana* plants treated with MeJA or SHAM. Anti‐CP, RSV CP antibody was used in western blot to detect the protein level of RSV CP. Ponceau S‐stained RuBisCO was used as the loading control. CP probe, the partial sequence of RSV CP gene labelled with digoxigenin was used in northern blot to detect the accumulation of viral RNA. Ethidium bromide‐stained total RNAs was used as the loading control in northern blot. The relative intensity of the blot signal quantified by ImageJ is shown above the lanes

To further confirm the defence role of the JA pathway against RSV on *N. benthamiana*, the tobacco rattle virus (TRV)‐induced gene silencing (VIGS) system was used to silence *COI1*, a key component of the JA pathway, before RSV inoculation. At 9 dpi of VIGS, expression of *COI1* in TRV:COI1‐infected plants was reduced to c.30% of the control level (TRV:00‐infected) and did not cause obvious changes in plant phenotype (Figure S1a,b). Plants were then mechanically inoculated with RSV. Compared to TRV:00, more serious RSV symptoms appeared on the systemically infected leaves of *COI1*‐silenced plants (Figure S1c). Northern and western blotting analyses showed that viral RNA and CP accumulation in leaves systemically infected with RSV was nearly 50% higher on *COI1*‐silenced plants than on the controls (Figure S1d).

The results above demonstrate that the JA pathway is induced by RSV infection and that it plays a defence role against RSV in both rice and *N. benthamiana*. Various viral proteins have been reported to change the expression of JA response genes (Diaz‐Pendon *et al*., 2007; Endres *et al*., 2010; Lewsey *et al*., 2010; Csorba *et al*., 2015). To detect which RSV protein was responsible for induction of the JA pathway, we expressed p2, p3, p4, pc4, and CP in leaves of *N. benthamiana* transiently by agroinfiltration (Li *et al*., 2019b) and detected their ability to induce JA. At 3 dpi of CP expression, *NbLOX*, *NbAOS*, and *NbOPR3* were up‐regulated to different extents. The expression level of *NbLOX* in zones expressing p2, *NbAOS* in zones expressing p4, and *NbAOC* in zones expressing pc4 were decreased, while the expression level of *NbOPR3* in zones expressing p3 was increased (Figures 3a and S2). The results imply that, of the five RSV proteins tested, CP is the major component responsible for inducing the JA pathway. Consistent with this, the JA content was enhanced significantly in zones expressing CP (Figures 3b and S3). In further experiments, transgenic rice plants were generated that expressed the CP gene driven by the cauliflower mosaic virus 35S promoter. Three genetically stable homozygous lines, CP#2‐1, CP#5‐3, and CP#9‐1, were used in these experiments (Figure 3c). Expression of CP was confirmed by western blot (Figure 3d). The expression levels of *Os13LOX*, *OsAOS*, and *OsOPR7* were increased in CP#2‐1 and CP#9‐1, and those of *Os13LOX* and *OsAOC* were increased in CP#5‐3 (Figure 3e). Moreover, in three transgenic rice lines expressing CP, JA content was significantly greater than in the wild type (Figure 3f). The results demonstrate that CP is an inducer of the JA pathway to activate plant defence against the virus, which is consistent with a recent paper by Yang *et al*. (2020).

**FIGURE 3:**
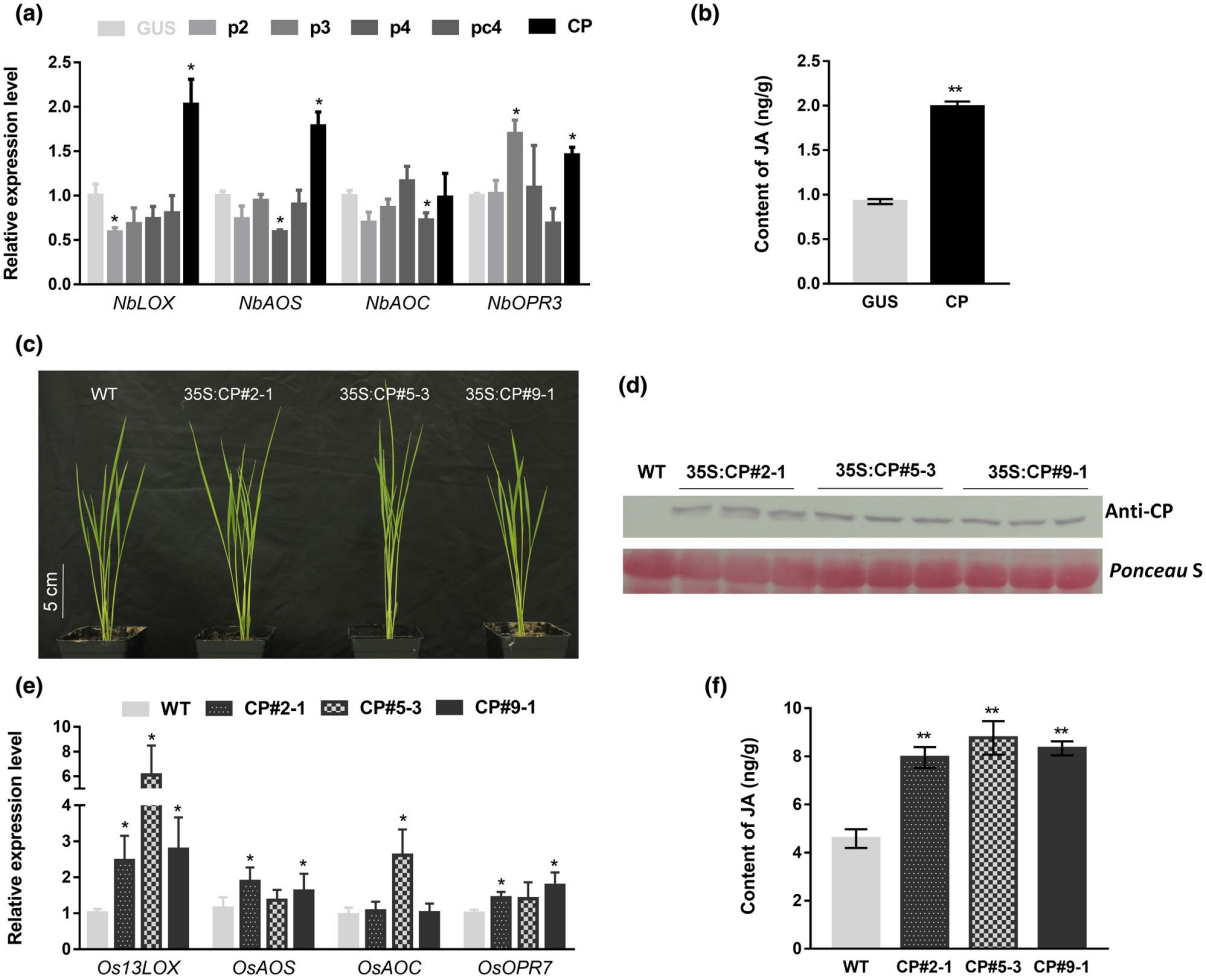
Rice stripe virus (RSV) coat protein (CP) is the major viral component responsible for induction of the jasmonic acid (JA) pathway. (a) Relative expression levels of JA pathway genes in leaves expressing RSV p2, p3, p4, pc4, or CP. (b) JA content was enhanced in leaves expressing RSV CP. (c) The phenotype of wild type (WT) (Nip) and CP transgenic rice (CP#2‐1, CP#5‐3, and CP#9‐1). Photographs were taken 15 days after germination. Scale bar = 5 cm. (d) Western blot confirmed the expression of CP in three transgenic lines. Total protein was extracted from rice seedlings 15 days after germination. Ponceau S‐stained RuBisCO was used as the loading control. (e) Relative expression levels of JA pathway genes in three transgenic rice lines expressing CP. (f) JA content was enhanced in transgenic lines. Bars represent the standard errors of the means from three biological repeats. A two‐sample unequal variance directional *t* test was used to test the significance of the difference (*p < .05, **p < .01)

RSV is transmitted by SBPHs in a persistent propagative manner. The JA pathway plays a key role in the tripartite interactions among plant, virus, and insect vectors (Wu and Ye, 2020). To explore the relationship between JA and SBPH performance, we examined the feeding preference of SBPHs on rice pretreated with MeJA or SHAM as described before (Zhou *et al*., 2009). MeJA pretreatment made plants significantly more attractive to SBPHs than the 0.1% ethanol‐treated controls at 4, 6, and 12 hr after the start of the experiment (Figure 4a). In contrast, SHAM pretreatment decreased SBPH attraction compared with the controls (Figure 4b).

**FIGURE 4:**
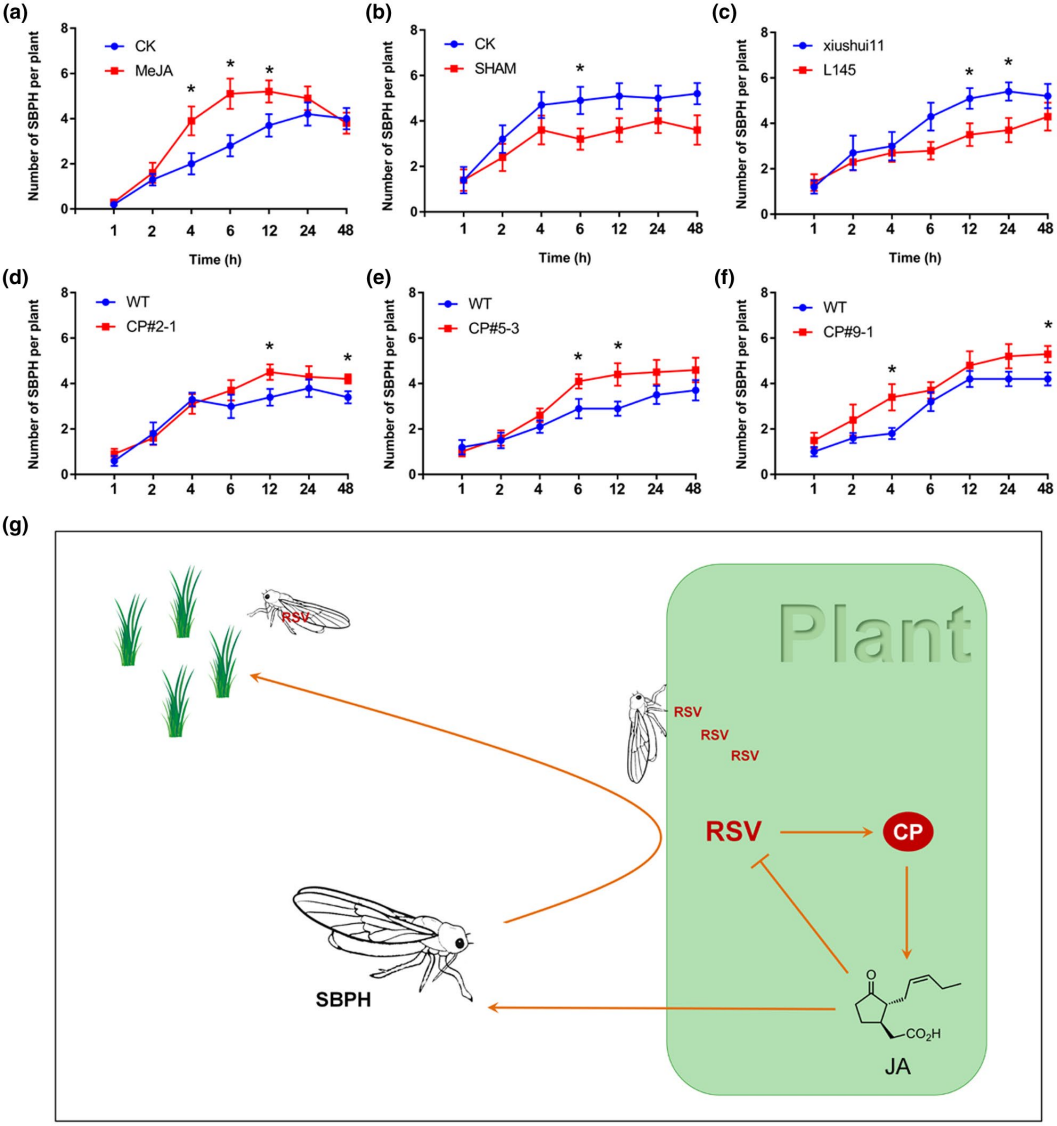
RSV coat protein (CP) increases the attractiveness of plants to small brown leafhopper (SBPH) vectors and depends on the jasmonic acid (JA) pathway. SBPH performance on (a) methyl jasmonate (MeJA)‐ and (b) salicylhydroxamic acid (SHAM)‐treated rice plants, with 0.1% ethanol‐treated plants as control (CK). (c) SBPH performance on *as‐lox* line L145‐1 and wild‐type (WT) (Xiushui11) plants. SBPH performance on CP transgenic lines CP#2‐1 (d), CP#5‐3 (e), and CP#9‐1 (f) and WT (Nip) plants. Mean number of SBPHs per plant (±*SEM*) on pairs of plants, 1–48 hr after 8–10 replicated plant pairs were exposed to 10 insects. A two‐sample unequal variance directional *t* test was used to test the significance of the difference (*p < .05). (g) A proposed model for the dual effect of JA in RSV infection. The JA pathway is induced by CP and activates the plant defence against RSV. Meanwhile, the enhanced JA also attracts SBPHs to feed and thus to benefit viral transmission

To confirm these findings, we also assessed the performance of SBPH on the *as‐lox* line L145‐1 and its wild‐type rice Xiushui11. In L145‐1 plants, the JA biosynthetic gene *OsHI‐LOX* (*13LOX*) is knocked down and therefore JA levels are significantly reduced (Zhou *et al*., 2009). L145‐1 plants were significantly less attractive to SBPH than Xiushui11 at 12 and 24 hr after the start of the experiment (Figure 4c).

As our results demonstrated that CP is the viral component responsible for induction of the JA pathway, we performed a feeding preference assay comparing CP‐transgenic and wild‐type (Nip) rice plants. All three individual CP‐transgenic lines (CP#2‐1, CP#5‐3, and CP#9‐1) were more attractive to SBPHs than the controls (Figure 4d–f), although there were some differences between the lines in the timing of this effect. These data demonstrate that the RSV CP increases the attractiveness of infected plants to SBPHs and that this is probably dependent on the JA pathway in this pathosystem.

The JA pathway plays crucial roles in plant defence against viruses. Defective JA perception aggravates viral infection, while extraneous application of MeJA can reduce viral infection (Wu and Ye, 2020). For example, *coi1‐13* mutant rice was sensitive to RBSDV infection, while foliar application of MeJA resulted in a significant reduction in RBSDV incidence (He *et al*., 2017). Also, JA treatment enhanced plant resistance to TMV, TSWV, and coinfection of PVX and PVY (Garcia‐Marcos *et al*., 2013; Zhu *et al*., 2014). Here, the results show that the JA pathway, which is induced during RSV infection, is also essential for plant defence against RSV, providing evidence of a defence role for JA in an RNA virus with an ambisense coding strategy.

Various viral proteins specifically induce the JA pathway. The ectopic expression of cucumber mosaic virus (CMV) 2b protein caused misregulation of JA‐responsive genes in *Arabidopsis* (Diaz‐Pendon *et al*., 2007; Lewsey *et al*., 2010; Csorba *et al*., 2015). HC‐Pro from turnip mosaic virus (TuMV) affected JA‐regulated gene expression, again in *Arabidopsis* (Endres *et al*., 2010). Both 2b and HC‐Pro are viral suppressors of RNA silencing, proteins that affect plant defence and development by significantly modifying gene expression. Here, we found that the transient expression of RSV CP caused changes in the expression of JA pathway genes, while the RNA silencing suppressor of RSV, p3, did not. The results demonstrate that, in RSV, the CP is the inducer of the JA pathway activating the plant defence against virus. Thus, RSV CP plays more roles in plant defence than we might otherwise have expected from a structural protein (Hayakawa *et al*., 1992; Lu *et al*., 2017).

It is generally assumed that JA‐dependent defences increase plant resistance to herbivorous insects, with a broad range of insects showing a preference for plants containing lower JA levels (Zhang *et al*., 2017). In contrast to this general phenomenon, our study demonstrated that SBPHs preferred to settle on rice plants with higher levels of JA, similar to the situation with another rice planthopper, *N. lugens* (Zhou *et al*., 2009; Dar *et al*., 2015). Planthopper infestation increases JA content in rice plants, which facilitates continuous planthopper feeding and attracts other planthoppers to select the preinfested plant (Cheng *et al*., 2001; Pan *et al*., 2018). Manipulation of plant defence through microbes has been reported in several hemipteran insect species. In the leafhopper *Macrosteles quadrilineatus*, JA biosynthesis was inhibited by insect‐transmitted bacteria, which improved the adaptability of insects to host plants (Sugio *et al*., 2011). In the whitefly *Bemisia tabaci*, begomoviruses also attenuate JA defence to promote vector performance (Li *et al*., 2019a). Our study found that the RSV CP was responsible for inducing the JA pathway and that SBPHs preferred to feed on rice plants overexpressing CP. We presume that RSV CP attracts SBPHs to feed by targeting the JA pathway and promoting virus spread.

Taken together, the results here demonstrate that CP is an inducer of the JA pathway that activates the plant defence against RSV while also attracting SBPHs to feed and thus to benefit viral transmission (Figure 4g). This is the first report of a function for JA in the tripartite interactions between RSV, its host, and its vector. Meanwhile, the mechanism of plant defence mediated by JA and the strategy used by RSV to escape the defence need further investigation.

## Data Availability

The data that support the findings of this study are available from the corresponding author upon reasonable request.
