# Supplementary figures and images for: Rice stripe virus coat protein induces the accumulation of jasmonic acid, activating plant defence against the virus while also attracting its vector to feed
Source: Mol Plant Pathol. 2020 Sep 24;21(12):1647–53. doi: 10.1111/mpp.12995 (PMC7694675; doi:10.1111/mpp.12995)

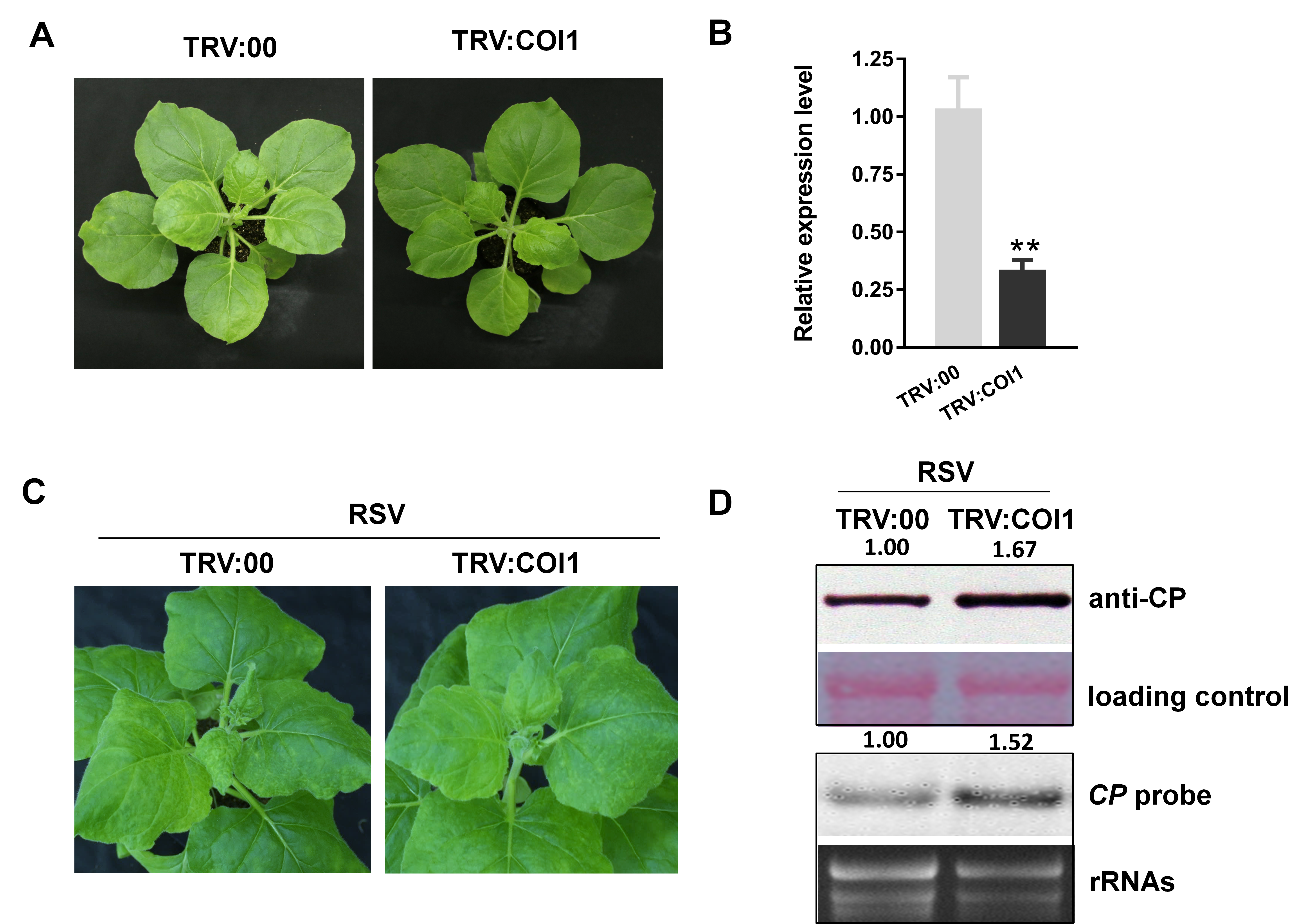

Supplement: Supplementary file 1 [file MPP-21-1647-s001.tif]

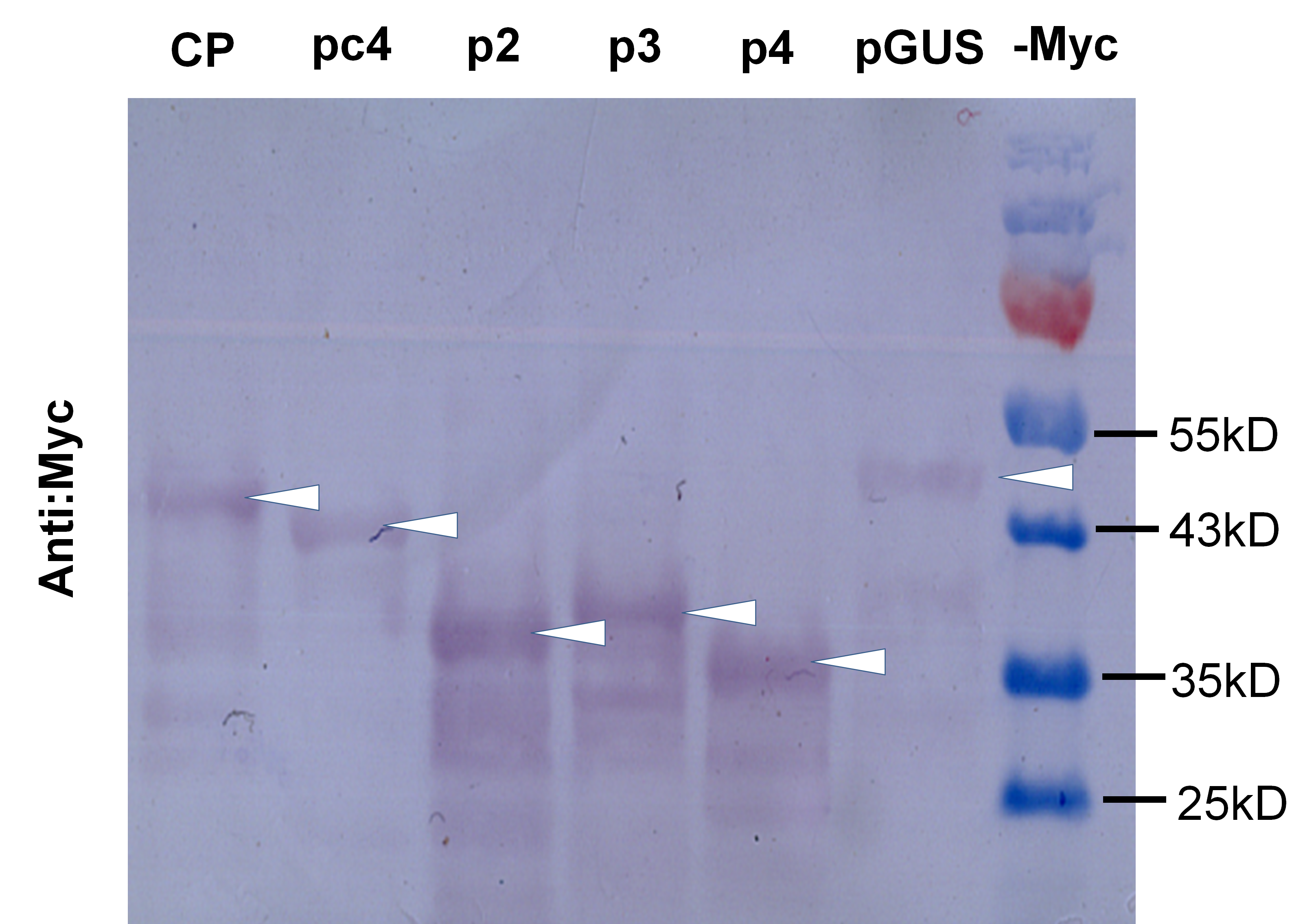

Supplement: Supplementary file 2 [file MPP-21-1647-s002.tif]

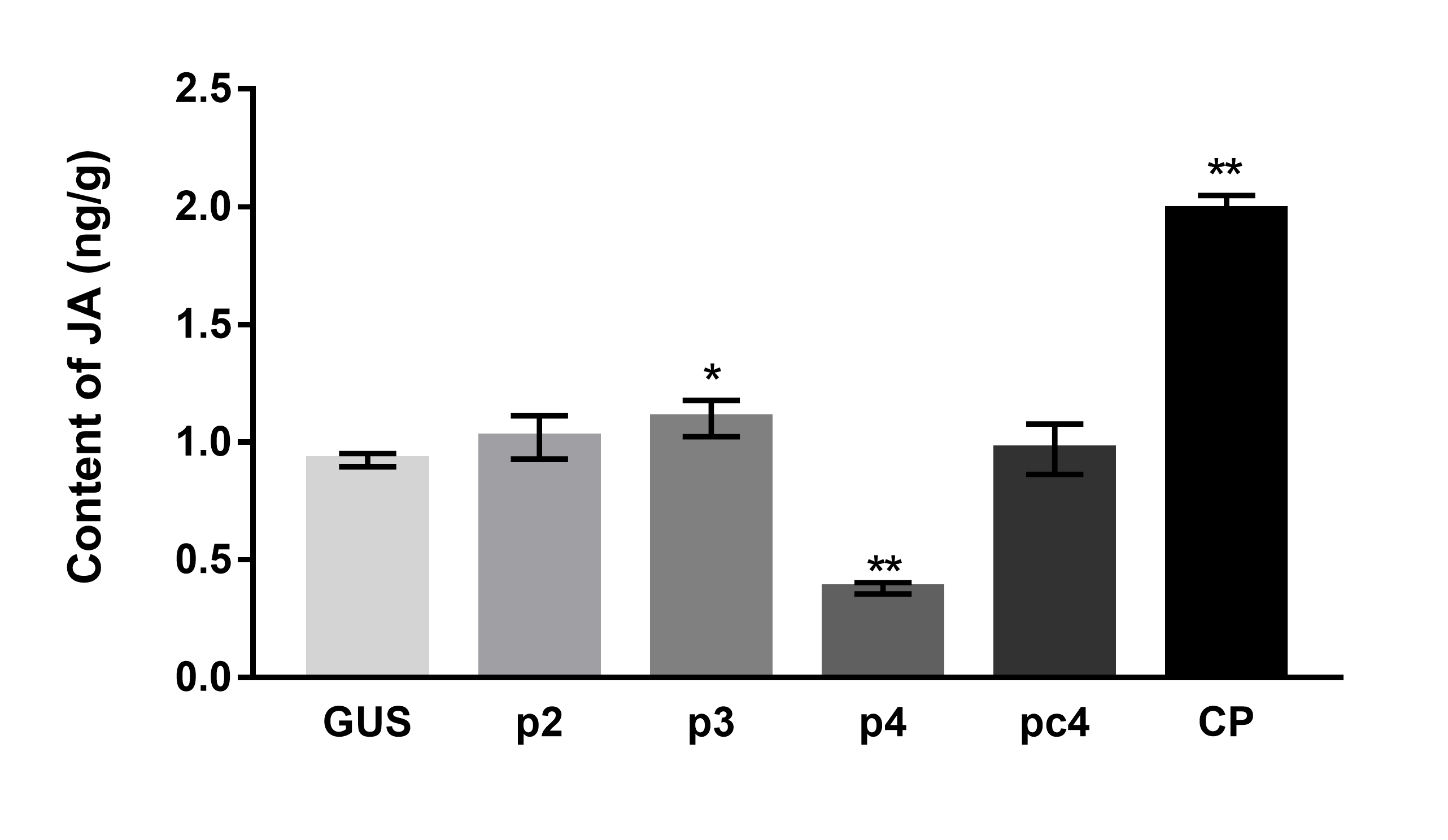

Supplement: Supplementary file 3 [file MPP-21-1647-s003.tif]
