# Supplementary material for: Rice stripe virus coat protein induces the accumulation of jasmonic acid, activating plant defence against the virus while also attracting its vector to feed
Source: Mol Plant Pathol. 2020 Sep 24;21(12):1647–53. doi: 10.1111/mpp.12995 (PMC7694675; doi:10.1111/mpp.12995)
Supplement: Supplementary file 4 [file MPP-21-1647-s004.docx]

**Experimental procedures**

**Plant materials and insect vector**

An as-lox line (antisense expression of OsHI-LOX) with its wild-type *O. sativa* L. japonica cultivar Xiushui11 and the CP transgenic lines (CP#2-1, CP#5-3 and CP#9-1, expressing the RSV CP driven by the cauliflower mosaic virus 35S promoter) with their wild-type counterpart *O. sativa* L. japonica cultivar Nipponbare were used in this study. Rice seedlings were grown in clay loam soil in a glasshouse under 16 h light (30 000-60 000 lux)/8 h dark photoperiod at 28℃ - 30℃ and 60 ± 5% relative humidity. *Nicotiana benthamiana* plants were grown in a glasshouse at 23°C - 25°C, with a 14 h light/10 h dark under well-watered conditions. The Virus-free SBPHs (*L. striatellus*) and viruliferous SBPHs (carrying RSV) were reared on Nipponbare seedlings in glass beakers enclosed with a piece of nylon mesh in a glasshouse at 25°C under artificial light.

**RSV inoculation**

RSV transmission via SBPH was done as described by Zhang (Zhang et al., 2020). Briefly, SBPH carrying RSV were transferred to rice seedlings (*O. sativa L*. japonica cultivar Nipponbare) at the 3- to 4-leaf stage (about three viruliferous insects per seedling). After 72 h, the insects were completely removed. *Nicotiana benthamiana* plants at the 5- to 6-leaf stage were mechanically inoculated with crude extracts from RSV-infected rice leaves, as described previously (Wu et al., 2013).

**Detection of JA content**

*Oryza sativa* and *Nicotiana benthamiana* from mock and RSV-inoculated plants were collected at 21 dpi and 14 dpi, respectively, ground in liquid nitrogen and then used for hormone extraction and analysis. For transient expression, agro-infiltrated *Nicotiana benthamiana* leaves were collected at 3 dpi. The samples (about 500 mg) were subjected to high‐performance liquid chromatography tandem mass spectrometry (HPLC‐MS/MS) analysis with JA‐type sample (Sigma‐Aldrich) according to a method previously described (Forcat et al., 2008). Three biological replicates were performed. The level of JA was measured by Zoonbio Biotechnology Co., Ltd.

**Vector construction**

To construct the transient expression vector, RSV CP was amplified and then introduced into the pCV vector (Lu et al., 2011). The inserted fragment was obtained by PCR using a pair of primers linked to *Xba* Ⅰ and *Sac* I sequences on either side. The pCV vector was digested with *Xba* Ⅰ and *Sac* I, then the digested vector and PCR product were treated with T4 DNA ligase. For expressing p2, p3, CP, p4 and pc4 tagged with Myc, the entire open reading frame (ORF) was fused with 4×Myc-tag and then introduced into the pCV vector. To suppress *NbCOI1*, a segment of *NbCOI1* was obtained by PCR and then ligated into the TRV-RNA2 vector as previously described (Liu et al., 2002). All primers are listed in Table S1.

**Exogenous application of MeJA and SHAM**

Methyl jasmonate (MeJA, Sigma-Aldrich) and Salicylhydroxamic acid (SHAM, Sigma-Aldrich) were dissolved in 100% ethanol to suitable concentrations as 1000× stock solutions and diluted with sterile distilled water. 0.1% ethanol was used as the control (CK). In experiments evaluating the effects of MeJA and SHAM on RSV infection, *N. benthamiana* were sprayed with 50 μM MeJA and 0.5 mM SHAM at 6 h before RSV inoculation. For RSV inoculation of rice, MeJA and SHAM were sprayed onto rice 6 h after feeding by viruliferous SBPH carrying RSV to avoid any effects of MeJA and SHAM on transmission of RSV by SBPH.

To assess the feeding preference of SBPH, 2- to 3-leaf stage rice seedlings were sprayed with 50 μM MeJA or 0.5 mM SHAM at 6 h before being exposed to SBPHs.

**Agroinfiltration of *Nicotiana benthamiana* plants**

For *Agrobacterium tumefaciens*-mediated transient expression, transient expression vectors were electroporated into *Agrobacterium tumefaciens* strain EHA105. Agroinoculation was carried out as described by Kapila (Kapila, 1997) .The cultures of *Agrobacterium tumefaciens* were diluted to OD_600_=1.0 in infiltration solution containing 10 mM MES, pH 5.6, 10 mM MgCl_2_ and 150 µM acetosyringone. *Agrobacterium tumefaciens* cultures were mixed well and incubated for 4 h at room temperature before infiltrating and were infiltrated onto the underside of leaves using a 1.0-mL syringe without a needle. *N. benthamiana* plants were inoculated at the 5- to 7-leaf stage. The inoculated plants were maintained in a growth chamber at 25 °C with 14h light/10h dark under well-watered conditions. The infiltrated leaves were detached 60-72 h post infiltration for the corresponding assays.

For virus-induced gene silencing of *NbCOI1* in *N. benthamiana*, TRV:COI1 was infiltrated into plants in combination with a TRV RNA1 vector as described before (Liu et al., 2002). A control infection consisted of the TRV RNA1 vector infiltrated in combination with an empty TRV2 vector (TRV:00), as previously described (Li et al., 2019). Plants agroinfiltrated for 12 days were used for RSV inoculation.

**Northern blot and quantitative RT-PCR**

Total RNA was isolated with Trizol (Invitrogen, Carisbad, California, USA) according to the manufacturer’s instructions. For northern blot, 5-10 µg of total RNA was separated on 1.5% formaldehyde agarose gels in 1×MOPS buffer, and blotted onto Hybond-N^+^ membranes (GE Healthcare, Chicago, IL, USA). Probes labelled with Digoxigenin (Roche, Basel, Switzerland) were prepared by amplifying RSV-CP fragments to detect RSV viral RNAs. The relative intensity of the blot signal quantified by ImageJ is shown above the lanes. The primers used are listed in Table S1.

First-strand cDNA was synthesized using the PrimeScript^TM^ RT reagent Kit with gDNA Eraser (Takara, Japan) with 1 µg total RNA. qRT-PCR was conducted on Roche LightCycler^®^480 Real-Time PCR System (Roche Diagnostics, Germany) using SYBR^®^ Green Realtime PCR Master Mix (Toyobo, Osaka, Japan) in accordance with the manufacturer’s instructions. The cycling conditions for the qRT-PCR were 95°C for 5 min, followed by 40 cycles of 95°C for 10 s, 60°C for 30 s, 72°C for 20 s. The results were analysed by the ΔΔC_T_ method (Livak & Schmittgen, 2001). *Oryza sativa* Actin (*OsActin*) or *Nicotiana benthamiana* Actin (*NbActin*) was used as the internal reference gene for analysis. At least three biological replicate samples were used. The primers used for qRT-PCR are listed in Table S1.

**Western blotting**

Total proteins of plant samples were extracted with 2 ml/g lysis buffer (100 mM Tris-HCl, pH 8.8, 60% SDS, 2 % β-mercaptoethanol), and then centrifuged at 12,000×g for 20 min at 4°C. Proteins were separated in a 12 % SDS-PAGE gel, transferred onto nitrocellulose (Amersham, Sweden) by wet electroblotting. Transferred proteins were detected with primary antibody to RSV CP (Rabbit polyclonal antibody), Myc-tag (Mouse monoclonal antibody; Transgene Biotech, Beijing, China) and an anti-rabbit (Sigma-Aldrich, St. Louis, Missouri, USA) or anti-mouse secondary antibody (Sigma-Aldrich, St. Louis, Missouri, USA), respectively. The antigen-antibody complexes were visualized using nitrotetrazolium blue chloride/5-bromo-4-chloro-3-indolyl phosphate (NBT/BCIP) buffer (Sigma-Aldrich, USA) under standard conditions. The relative intensity of the blot signal quantified by ImageJ is shown above the lanes.

**Generation of CP transgenic lines**

To generate transgenic plants expressing CP, the full-length ORF of CP was cloned into the pCV vector downstream of the CaMV 35S promoter. This vector was used for transforming *Oryza sativa* L. japonica cultivar Nipponbare (Nip) using an Agrobacterium-mediated transformation procedure (Hiei et al., 1994). These transgenic plants were confirmed by western blot assays. For this study, three T_2_ lines, CP#2-1, CP#5-3 and CP#9-1 (Figure S3), and WT (Nip) plants were used.

**SBPH performance measurement**

The colonization preference of SBPH was performed as described previously (Zhou et al., 2009). In brief, pots with two plants (a MeJA-treated or SHAM-treated plant and CK-treated plant, an as-lox line plant and a WT (Xiushui11) plant, a CP transgenic line plant and a WT (Nip) plant) were individually confined within plastic cages (diameter 10 cm, height 20 cm). The container was then covered with 200-mesh plastic screen to constrain SBPH. Ten third- to fourth-instar nymphs were released in the center of the container. The number of SBPH on each plant was calculated at 1, 2, 4, 6, 12, 24 and 48 h after the release of SBPH. The experiment was repeated 8-10 times.

Table S1 Primers used in the experiments

| **Name** | **sequences** | **Description** |
| --- | --- | --- |
| *OsActin* RT(+) | 5'-atgtcgcaatccaggctgtt-3' | primer as internal control used for qRT-PCR |
| *OsActin* RT(-) | 5'-accggaggatagcatgagga-3' |  |
| *Os13LOX* RT(+) | 5'-atggccggaacaaggatag-3' | primer used for qRT-PCR |
| *Os13LOX* RT(-) | 5'-tcagatggatgtgctgttgg-3' |  |
| *OsAOS*2 RT(+) | 5'-caatacgtgtactggtcgaatgg-3' | primer used for qRT-PCR |
| *OsAOS*2 RT(-) | 5'-aaggtgtcgtaccggaggaa-3' |  |
| *OsAOC* RT(+) | 5'-agcacatgtagccaccatcc-3' | primer used for qRT-PCR |
| OsAOC RT(-) | 5'-cgatacgcattgatggacgga-3' |  |
| *OsOPR7* RT(+) | 5'-ttgggttggaagcagtggag-3' | primer used for qRT-PCR |
| *OsOPR7* RT(-) | 5'-ggatcgggcgtgtagaatgt-3' |  |
| *NbActin* RT(+) | 5'-ggactctggtgatggtgtca-3' | primer as internal control used for qRT-PCR |
| *NbActin* RT(-) | 5'-gaacatgtaaccacgctcgg-3' |  |
| *NbLOX* RT(+) | 5'-attaacgtgggccatgcaga-3' | primer used for qRT-PCR |
| *NbLOX* RT(-) | 5'-gccgaaattcagcgaagcat-3' |  |
| *NbAOS* RT(+) | 5'-gcgaacccagttgagactaag -3' | primer used for qRT-PCR |
| *NbAOS* RT(-) | 5'-atcttcatcccgccaaag-3' |  |
| *NbAOC* RT(+） | 5'-acagcttctacttcggcgat-3' | primer used for qRT-PCR |
| *NbAOC* RT(-） | 5'-ggcagatccggaataccctt-3' |  |
| NbOPR3 RT(+) | 5'-tttcacacaggagcaagaag-3' | primer used for qRT-PCR |
| NbOPR3 RT(+) | 5'-atgagttccatctggcattag-3' |  |
| NbCOI1 RT(+) | 5'-cactggtcgggatctcttgg-3' | primer used for qRT-PCR |
| NbCOI1 RT(+) | 5'-taggcaagtatatgggctgg-3' |  |
| NbCOI1 VIGS(+) | 5'-cctgcaggatctaccccttgataatgg-3' | forward primer for TRV:COI1 by PstI |
| NbCOI1 VIGS(+) | 5'-cctgcagaggccttcatcggattcccc-3' | reverse primer for TRV:COI1 by PstI |
| RSV-CP probe F | 5'-aggcaatcaatgacatctcc-3' | primer used for Northern blot |
| RSV-CP probe R | 5'-atctctcacaaagccagtgc-3' |  |
| CP1 XbaI (+) | 5'-ttctaga atgggcacca acaagccagc-3' | forward primer for pCV:CP by XbaI |
| CP322 SacI(-) | 5'-ggagctc ctagtcatct gcaccttct-3' | reverse primer for pCV:CP by SacI |
| p2 LIC（+） | 5'-cgacgacaagaccgtcaccatggcattactcctcttcaa-3' | forward primer for pCV:p2-Myc by lic |
| p2 LIC（-） | 5'-gaggagaagagccgtcgcattagaatagggcactcat-3' | reverse primer for pCV:p2-Myc by lic |
| p3 LIC（+） | 5'-cgacgacaagaccgtcaccatgaacgtgttcacatcgtc-3' | forward primer for pCV:p3-Myc by lic |
| p3 LIC（-） | 5'-gaggagaagagccgtcgcagtacagctagagagctgc-3' | reverse primer for pCV:p3-Myc by lic |
| p4 LIC（+） | 5'-cgacgacaagaccgtcaccatgcaagacg tacaaaggac-3' | forward primer for pCV:p4-Myc by lic |
| p4 LIC（-） | gaggagaagagccgtcgtgttttgtgt agaagaggtt-3' | reverse primer for pCV:p4-Myc by lic |
| CP LIC（+） | 5'-cgacgacaagaccgtcaccatgggcacca acaagccagc-3' | forward primer for pCV:CP-Myc by lic |
| CP LIC（-） | gaggagaagagccgtcggtcatct gcaccttctg c-3' | reverse primer for pCV:CP-Myc by lic |
| pc4 LIC（+） | 5'-cgacgacaagaccgtcaccatggctttgtctcgacttttg-3' | forward primer for pCV:pc4-Myc by lic |
| pc4 LIC（-） | 5'-gaggagaagagccgtcgcatgatgacagaaacttcag-3' | reverse primer for pCV:pc4-Myc by lic |
| pGUS LIC（+） | 5'-cgacgacaagaccgtcacc atgttacgtcctgtagaaac-3' | forward primer for pCV:pGUS-Myc by lic |
| pGUS LIC（-） | 5'-gaggagaagagccgtcggtccgcatcttcatgacgac-3' | reverse primer for pCV:pGUS-Myc by lic |

**References**

Forcat S, Bennett MH, Mansfield JW, Grant MR, 2008. A rapid and robust method for simultaneously measuring changes in the phytohormones ABA, JA and SA in plants following biotic and abiotic stress. *Plant Methods* **4**, 16.

Hiei Y, Ohta S, Komari T, Kumashiro T, 1994. Efficient transformation of rice (Oryza sativa L.) mediated by Agrobacterium and sequence analysis of the boundaries of the T-DNA. *Plant J* **6**, 271-82.

Kapila J DRR, Van Montagu M, Angenon G, 1997. An Agrobacteriummediated transient gene expression system for intact leaves. *Plant Science* **122**, 101-8.

Li S, Zhao J, Zhai Y*, et al.*, 2019. The hypersensitive induced reaction 3 (HIR3) gene contributes to plant basal resistance via an EDS1 and salicylic acid-dependent pathway. *Plant J* **98**, 783-97.

Liu Y, Schiff M, Dinesh-Kumar SP, 2002. Virus-induced gene silencing in tomato. *Plant J* **31**, 777-86.

Livak KJ, Schmittgen TD, 2001. Analysis of relative gene expression data using real-time quantitative PCR and the 2(-Delta Delta C(T)) Method. *Methods* **25**, 402-8.

Lu Y, Yan F, Guo W*, et al.*, 2011. Garlic virus X 11-kDa protein granules move within the cytoplasm and traffic a host protein normally found in the nucleolus. *Mol Plant Pathol* **12**, 666-76.

Wu G, Lu Y, Zheng H, Lin L, Yan F, Chen J, 2013. Transcription of ORFs on RNA2 and RNA4 of Rice stripe virus terminate at an AUCCGGAU sequence that is conserved in the genus Tenuivirus. *Virus Research* **175**, 71-7.

Zhang H, Li L, He Y*, et al.*, 2020. Distinct modes of manipulation of rice auxin response factor OsARF17 by different plant RNA viruses for infection. *Proc Natl Acad Sci U S A* **117**, 9112-21.

Zhou G, Qi J, Ren N*, et al.*, 2009. Silencing OsHI-LOX makes rice more susceptible to chewing herbivores, but enhances resistance to a phloem feeder. *Plant J* **60**, 638-48.
